# Evaluation of sisal fiber and aluminum waste concrete blend for sustainable construction using adaptive neuro-fuzzy inference system

**DOI:** 10.1038/s41598-023-30008-0

**Published:** 2023-02-16

**Authors:** Chima Dike Agor, Elvis Michael Mbadike, George Uwadiegwu Alaneme

**Affiliations:** 1 Department of Civil Engineering, Michael Okpara University of Agriculture, Umudike, P. M. B. 7267, Umuahia, 440109 Abia State Nigeria; 2 Department of Civil Engineering, Kampala International University, Kansanga, Uganda

**Keywords:** Civil engineering, Structural materials

## Abstract

This research study presents evaluation of aluminum waste-sisal fiber concrete’s mechanical properties using adaptive neuro-fuzzy inference system (ANFIS) to achieve sustainable and eco-efficient engineering works. The deployment of artificial intelligence (AI) tools enables the optimization of building materials combined with admixtures to create durable engineering designs and eliminate the drawbacks encountered in trial-and-error or empirical method. The features of the cement-AW blend's setting time were evaluated in the laboratory and the results revealed that 0–50% of aluminum-waste (AW) inclusion increased both the initial and final setting time from 51–165 min and 585–795 min respectively. The blended concrete mix's flexural strength tests also show that 10% sisal-fiber (SF) substitution results in a maximum flexural strength of 11.6N/mm^2^, while 50% replacement results in a minimum flexural strength of 4.11N/mm^2^. Moreover, compressive strength test results show that SF and AW replacements of 0.08% and 0.1%, respectively, resulted in peak outcome of 24.97N/mm^2^, while replacements of 0.5% and 0.45% resulted in a minimum response of 17.02N/mm^2^. The ANFIS-model was developed using 91 datasets obtained from the experimental findings on varying replacements of cement and fine-aggregates with AW and SF respectively ranging from 0 to 50%. The ANFIS computation toolbox in MATLAB software was adopted for the model simulation, testing, training and validation of the response function using hybrid method of optimization and grid partition method of FIS at 100 Epochs. The compressive strength behavior is the target response, and the mixture variations of cement-AW and fine aggregates-SF combinations were used as the independent variables. The ANFIS-model performance assessment results obtained using loss function criteria demonstrates MAE of 0.1318, RMSE of 0.412, and coefficient of determination value of 99.57% which indicates a good relationship between the predicted and actual results while multiple linear regression (MLR) model presents a coefficient of determination of 82.46%.

## Introduction

Increase in population, urban growth, and commercialization have been observed to pose a direct impact on rising levels of energy demand and material utilization and, therefore bring about generation of solid wastes^1,2^. The notion of sustainable development offers one potential remedy for this issue which refers to a course of development that protects natural resources and builds assets in a way that ensures meeting current demands of the people without compromising those of the future generations^3,4^. One of the few always ever-present concerns is the issue of sustainable and ecofriendly infrastructural development because it is crucial to contemporary society. Given that the current notion is relevant to all types of human activity, the application field for sustainability is almost limitless^5,6^. The need to determine viable alternatives to the well-known concrete ingredients to achieve sustainability and eco-efficient environment has led to exploitation of solid-waste and the derivatives^7^. These solid waste materials handling and poor management around the world and more especially in the developing countries have contributed to severe environmental degradation^8,9^. So, introduction of supplementary-cementitious-materials (SCM) which possesses alumina-silicates contents is helpful towards achieving sustainability and ecofriendly concrete materials through reduction of greenhouse gases emissions^10,11^.

Utilization or recycling of solid-wastes in production of concrete is also essential towards waste management and involves complex analysis of the factor levels to achieve desired response as regards mechanical and resilience behavior^12^. By investigating and evaluating the efficacy of additional locally accessible materials that are classified as either industrial or agricultural wastes, some researchers have attempted to reduce the total cost of building materials^13,14^. Concrete's compressive strength and durability properties are improved by the addition of solid-waste derivatives with alumina-silicate content^15^. Ayat et al*.*^16^ researched on the appropriate ratio of supplementary cementing materials (SCMs), including limestone filler (LF), which was mixed with Portland cement, resulting to environmental, economic and technological advantages. ANN was deployed in the study which were trained using the Tan-sigmoid activation function and the feed-forward back-propagation optimization technique and the results showed strong correlations that exceeded 97%.

Utilization of fibers in concrete helps to improve its post-cracking and ductility characteristics to achieve fiber reinforced concrete. This improved ductility behavior is due to the insertion of fibers that can efficiently transmit tensile stresses throughout a damaged region, hence narrowing the cracks^15^. Fiber reinforced-concrete is a unique and an important area of research given its significance whereby concrete’s properties such as tensile strength are enriched with fibers which is also devoid of compromise on the compressive strength properties. Concrete under impact force fails extensively as a consequence of its brittle nature^16^. Therefore, addition of fibers intensifies its many engineering properties such as toughness, flexural strength, impact, resistance to fatigue loads, and thermal shocks. The use of natural fiber reinforced composites is now considered as a new perspective in the construction industry^17^. Concrete materials possess tough compression characteristics but weak and fragile to resist series of tension forces and hence, there is need to provide support or reinforcement to it. In many instances, steel materials reinforcement was utilized; but a significantly large number of researches are in headway to discover a substitute to steel through the utilization of natural fibers^18^.

According to earlier studies, sisal-fiber reinforced concrete has improved the concrete's performance, including its workability, strength and durability^19^. Sisal-fiber has the potential to be employed as admixture and offer good properties especially the compressive, tensile as-well-as flexural-strength, but they are influenced by the fiber-length^20^. Dhumal et al*.*^21^, conducted research to compare conventional concrete and sisal-fiber reinforced-concrete with varying length to assess the influence of the fiber-length in concrete. Volume fraction and aspect ratio are crucial batching criteria used to describe fiber. The aspect ratio (l/d) is the proportion of a fiber's length to its diameter which ranges from 30–150 and plays a crucial part in improving the mechanical behavior of the concrete^13,22^. Conversely, the volume of fiber in a concrete matrix is known as volume fraction. However, current research suggests that fibers are introduced through weight ratio of cement. According to some studies, the ideal fiber content by cement mass is 1%; any higher additions cause a loss in the concrete's compressive and split tensile strengths, respectively, only for tiny modulus fibers^14,23^.

Artificial-intelligence (AI) or machine-learning refers to any system that presents features concomitant with a human mind, such as learning and problem-solving. AI copycats’ human intelligence and is used in expert, knowledge-based, and robot systems^24^. Neural networks are used as appropriate tools of pattern matching and can have their weights automatically adjusted to have the response function optimized, despite their inability to infer how to arrive at certain decisions^25^. On the other hand, fuzzy logic overcomes this limitation by inferring how to arrive at a certain judgment but is unable to educate itself and learn autonomously. As a consequence, the concurrent use of both techniques might serve as complements with the aim of resolving the many problems that non-hybrid soft computing techniques have^26,27^. Among these, the Adaptive Neuro-Fuzzy Inference System (ANFIS) is one of the best possible modeling tools, having many applications in several fields of inquiry and study. Jang introduced ANFIS, which provides connections between neural networks and fuzzy logic for modeling complex and dynamic systems^28^. Its functional set of rules is derived from a combination of the least-squares and back-propagation algorithm that serves as an indicator to adjust the parameters and output of the fuzzy inference system (FIS). The If–then rules are also implemented in the ANFIS data processing mechanism through generating a mapping between the variables of input and output^29^.

In order to obtain a specified design strength and other desired features, concrete-mix-design comprises the evolution of establishing the quantities of the different elements of concrete. Although the assessment of the mixture-response is often an empirical process based on specific design codes, statistical methodologies and response surface methods have been applied^30,31^. In recent times, smart intelligent models are now developed for the optimization of the response parameter to obtain the mixture ratio with maximum response at minimum number of trials^32,33^. Alaneme et al*.*^34^ researched on ANFIS optimization of compressive-strength performance of periwinkle shell (PWS) and rice-husk-ash (RHA) concrete. Sixty-two (62) datasets derived in the laboratory outcomes were employed to develop the model. The ANFIS-model performance evaluated through loss-function-parameters presented MSE of 0.3906, RMSE of 0.625, MAE of 0.2244, and coefficient of determination (r^2^) of 98.67%. Also, Nataraja et al*.*^35^, proposed a fuzzy-neuro model for concrete component design. In order to measure out the necessary amounts of fine and coarse aggregates, cement and water for a concrete mix's required mechanical properties, 5-layered FIS and three-layered-ANN were hybridized. It was determined that the model performs adequately when compared to traditional approaches, but provides slightly lower contents than the latter within a tolerable range.

The research study overall idea is to integrate industrial residue such as aluminum waste (AW) with appreciable pozzolanic comportment and agro waste like sisal fiber to partially replace the core concrete components of cement and fine aggregates respectively, from 0–50%. The essence of this exercise is to recycle solid industrial and agro wastes so as to achieve sustainable, eco-efficient and green concrete with enhanced mechanical and durability performance. This experimental investigation is aimed at the deployment of Soft-computing technique (ANFIS) to appraise the mechanical response to facilitate the determination of the optimal combination of the green fiber-reinforced concrete ingredients.

## ANFIS

Jang's proposed Adaptive Neuro-fuzzy inference system (ANFIS) provides a realistic procedural environment that combines Neural-Network and Fuzzy-logic philosophies to aid the fuzzy-model research and understanding of details from provided clusters of data^36,37^. It is a network of directed linkages and nodes that can be learned from and adjusted, and it produces learning rules related to membership function requirements that can be obtained from datasets, ensuring proper statistical inference of data^38^. ANFIS imitates the neural network's capacity to learn in order to generate appropriate MF and similar if–then rules of fuzzy sets from a given input–output model variables connection. Because it joins in together neural-networks and fuzzy-logic codes, it is capable of capturing the advantages of the two in a one outline. Its interpretation structure matches to a group of fuzzy-IF–THEN rules with adaptability and potential to round-up non-linear tasks^39^.

### Design network architecture of ANFIS

**Figure 1:**
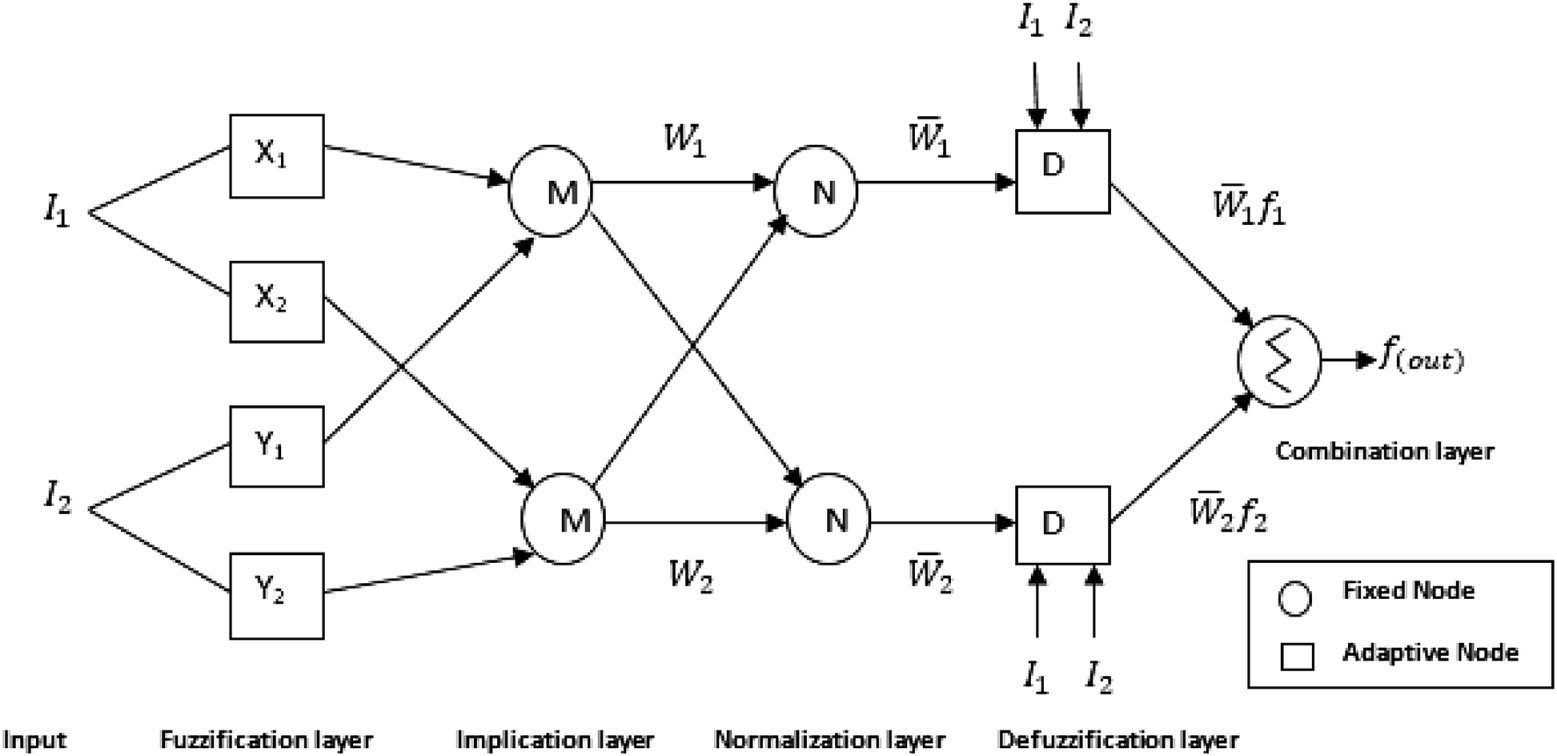
ANFIS architecture.

Figure 1 depicts the structural and functional layout of ANFIS, which uses 5-layers to build the smart model with each layer associated with several node functions. Equations 1–2 provide the fuzzy-if–then-rules for Sugeno fuzzy first order as well as the ANFIS-model fuzzy perceptive tool for two inputs ${\text{I}}_{{1{ }}} \;{\text{and}}\;{\text{I}}_{{2{ }}}$. where A_i_ and B_i_ are the membership function parameters, while P_i_, q_i_ and r_i_ are the learnable parameters of the output variables^40,41^.

1 $${\text{First}}\;{\text{rule:}}\;{\text{if}}\;{\text{I}}_{1} = {\text{A}}_{1} \;{\text{and}}\;{\text{I}}_{2} = {\text{B}}_{2} ,\;{\text{then}}\;{\text{f}}_{1} = {\text{p}}_{1} {\text{I}}_{{1{ }}} + {\text{q}}_{1} {\text{I}}_{2} + {\text{r}}_{1}$$

2 $${\text{Second}}\;{\text{rule:}}\;{\text{if}}\;{\text{I}}_{1} = {\text{A}}_{2} \;{\text{and}}\;{\text{I}}_{2} = {\text{B}}_{2} ,\;{\text{then}}\;{\text{f}}_{2} = {\text{p}}_{2} {\text{I}}_{{1{ }}} + {\text{q}}_{2} {\text{I}}_{2} + {\text{r}}_{2}$$

The five isolated strata of the ANFIS-framework are explained thus;

***Layer 1:*** The first Fuzzy layer possesses adaptive nodes with node-functions that convert inputs into linguistic labels of fuzzy set in order to determine data membership values. This stratum is in charge of the input data-fuzzification, in which the crisp data are plotted into attributes ranging from 0 to 1, allowing the degree of relatedness of the constraints to be calculated. Similarly, the membership function values required for each ith node are anticipated as indicated in Eq. (3).^42,43^.

3 $${\text{o}}_{{\text{i}}}^{1} = {\upmu }_{{{\text{{\rm A}i}}}} ({\text{I}}_{{\text{i}}} )\;{\text{for}}\;{\text{i}} = 1,2$$

where ${\text{I}}_{{\text{i}}}$ is the input variable for the ith node and ${\text{A}}_{{\text{i}}}$ the linguistic variables associated to the node function and ${\upmu }({\text{I}}_{{\text{i}}} )$ is the derived membership function.

***Layer 2:*** This layer possesses fixed nodes which computes the product of all input signals as the associated firing strength. The outcome of the firing-strength (signal) coming as stated in Eq. (4) is used to get the rule-matching-factor ${\text{W}}_{{\text{i}}}$, which is the firing-strength^44,45^.

4 $${\text{o}}_{{\text{i}}}^{2} = {\upmu }_{{{\text{{\rm A}i}}}} ({\text{I}}_{{2}} ) \times {\upmu }_{{{\text{Bi}}}} ({\text{I}}_{{2}} ) = {\text{W}}_{{\text{i}}} {;}\;{\text{for}}\;{\text{i}} = 1,2.$$

***Layer 3:*** normalization of MF parameters is realized in this layer. The regularization is achieved by dividing the layer node’s ith signal computed by the sum total of all signal rules as shown in Eq. (5)^38,46^.

5 $${\text{o}}_{{\text{i}}}^{3} = \frac{{{\text{W}}_{{\text{i}}} }}{{\sum {\text{W}}_{{\text{i}}} }} = \frac{{{\text{W}}_{{\text{i}}} }}{{{\text{W}}_{{1 + {\text{W}}_{2} }} }} = {\overline{\text{W}}}_{{\text{i}}} ;\;{\text{for}}\;{\text{i}} = 1,2.$$

***Layer 4:*** Sugeno first order polynomial was applied in the layer to obtain relationship among the input–output parameter. The node function is adjustable as described in Eqs. (6, 7).

6 $${\text{o}}_{{\text{i}}}^{4} = {\overline{\text{W}}}_{{\text{i}}} \times {\text{f}}_{{\text{i}}} {;}\;{\text{for}}\;{\text{i}} = 1,2.$$

7 $${\text{o}}_{{\text{i}}}^{4} = \overline{{{\text{W}}_{{\text{i}}} }} \left( {{\text{p}}_{{\text{i}}} {\text{I}}_{1} + {\text{q}}_{{\text{i}}} {\text{I}}_{2} + {\text{r}}_{{\text{i}}} } \right)$$

where f_1_ and f_2_ are the if–then fuzzy results presented in Eqs. (1, 2), where (${\text{p}}_{{\text{i}}}$, ${\text{q}}_{{\text{i}}}$ and ${\text{r}}_{{\text{i}}}$) are set of adaptive parameters referred to as the consequent variables^47,48^.

***Layer 5:*** This layer, often referred to as the defuzzification layer, is made up of a single solitary node that creates the total of all signals that are received by succeeding nodes, with the end result typically being a single value as shown in Eq. (8)^43,49^.

5 $${\text{o}}_{{\text{i}}}^{5} = \mathop \sum \limits_{{\text{i}}} {\overline{\text{W}}} \times {\text{f}}_{{\text{i}}} = \frac{{\mathop \sum \nolimits_{{\text{i}}} {\text{W}}_{{\text{i}}} {\text{f}}_{{\text{i}}} }}{{\mathop \sum \nolimits_{{\text{i}}} {\text{W}}_{{\text{i}}} }}$$

## Method and materials

### Materials

#### Portland cement

In this investigation, Dangote cement (42.5 grade) with a 30-percent normal consistency that complied with CEM II standards as stated in Nigerian Industrial Standard (NIS) 444–1 requirements, proportions, and compliance indicators was employed^50^.

#### Water

Water is a crucial component that has an impact on the rheological and mechanical characteristics of concrete. For this investigation, clean, drinkable water was used, and it complies with ASTM C1602-12 specifications for water to be used in concrete mixtures^51^.

#### Sisal fiber

Locally available sisal fiber from Akwa Ibom state were processed manually and used for the experimental investigation. Sisal fiber was chopped into 20-25 mm pieces and used for the investigation. Using a Hounsfield tensometer, the sisal fiber's tensile strength was measured. To guarantee durability, the fibers were given a silica fume treatment by being submerged in a slurry before being added to the concrete and given 13 min to dry.

#### Aluminum waste

The Aluminum waste was obtained from Aluminum Extrusion Industry (ALEX), Inyishi in Ikeduru L.G.A., Imo State, Nigeria. It is produced by heating aluminum scraps at a temperature of 1980 °C in a furnace. The waste is then sieved through a 150 μm sieve size so as to obtain the particles of waste in a finely divided state in accordance with ASTM C618–78 specification^52^.

#### Fine and coarse aggregates

The studies utilized sand taken from a clean river bed in Cross River State, Nigeria as the fine aggregates. It was made in accordance with BS EN 12,620 requirements and sieved via a 2.36 mm standard size^53^. The experimental investigation also employed clean, well-graded crushed granite with a maximum particle size of 20 mm that was procured from Abia State for the coarse aggregates, and it likewise adhered to ASTM C125-16^54^.

### Methods

#### Compressive strength test

A conventional batching concrete mix of 1:1.5:3 and a w/c of 0.55 were the mixture design used, which included the replacement of fine aggregate and cement in the green fiber concrete mixture with sisal fiber and aluminum waste from 0 to 50%. The concrete mixture materials were then completely mixed with water to achieve a homogeneous mixture before placement and compaction in the 150 mm × 150 mm × 150 mm cubic mould dimensions. After 24 h, the concrete samples were placed in a curing tank at room temperature for 28 days. Before crushing, it is weighed to assess its compressive strength, which is computed using Eq. (9)^55,56^.

9 $${\text{CS}} = \frac{{Failure\;load\; \left( {\text{N}} \right)}}{{cross}{-}{sectional\;area\;of\;concrete\; cube\; \left( {{\text{mm}}^{2} } \right)}}$$

#### Flexural strength test

Concrete beam moulds measuring 100 mm by 100 mm by 400 mm were used to test flexural strength. The concrete beams were cast and hydrated at 28 days with mixing proportion of 1:1.5:3 and water-cement fraction of 0.55 for the mixture design comprising replacement of cement and fine aggregate in the green fiber concrete mixture with sisal fiber and Aluminium waste correspondingly from 0–50%. Three specimens were cast and cured after 28 days. After 28 days of curing, the three specimens for each combination were crushed and the average flexural strength was measured using Eq. (10) in accordance with ASTM C1161-02c(2008)e1^57^.

10 $$\sigma = \frac{FL}{{bd^{2} }}$$

#### Setting time test

The Vicat apparatus is used to achieve the final and initial setting of the freshly blended mixture paste in compliance with CEN-EN 196-3 specifications^58^. During the experiment, 400 g of cement-Aluminum varying fractions were mixed with 0.85P of water to form a paste blend where P represents the paste normal consistency. Once the vicat mold is filled with the plastic mixture, it is gently set on the vicat equipment. The initial setting pin is fitted to the vicat apparatus to calculate the initial setting time before the vicat mold with the mixed paste is placed on it. The vicat apparatus's calibration mark is pasted to a ± 5 mm distance in millimeters, and the starting pin is dropped there to determine the setup time. After that, the amount of time that passed between the addition of water to the cement and the period in minutes the pin was dropped is noted, leaving a mark on the apparatus of ± 5 mm, indicating the first setting time. Immediately after the first setting time reading is acquired, the apparatus is connected, and this pin is used to determine the final setting time. The inner pin was allowed to descend freely, and the final setting time was recorded when it left no marks on the paste. When the inner final setting pin leaves a mark on the paste, the final setting time is then recorded, beginning when the cement was first mixed with water^59^.

### Flowchart of the ANFIS modelling algorithm

**Figure 2:**
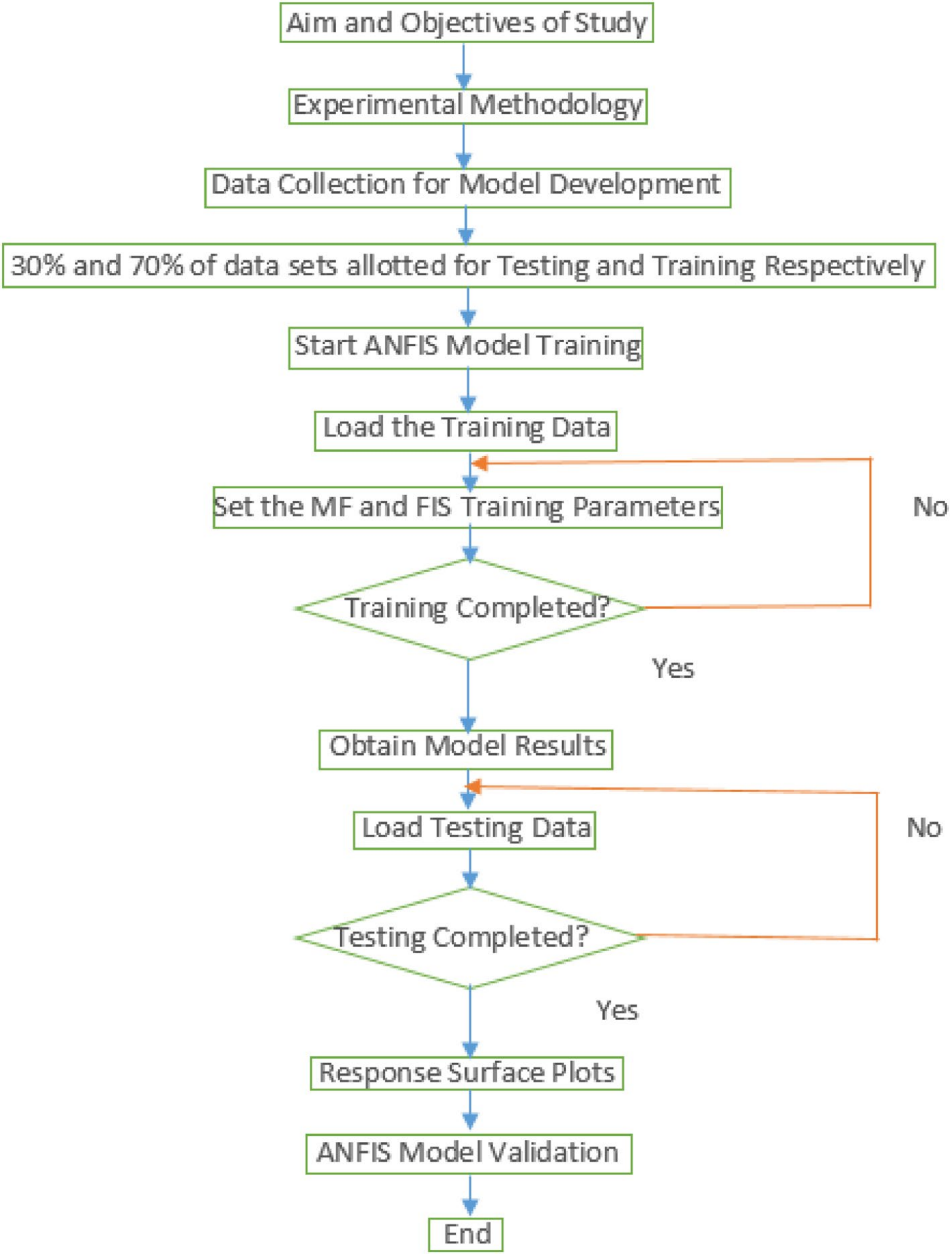
Research study flowchart.

Following the extraction of experimental findings from the laboratory, the mixed design data and the accompanying answers created were logically sorted to provide the needed variables for model construction. The datasets are separated into two pieces of 30% and 70% for testing and training the model, accordingly. Following training, the smart model generated learns to generalize the experimental data that it has been given in order to make correct predictions. Figure 2 depicts the methodology's algorithm flowchart. providing the sequence of events or activities for the research study that begins with the definition of study goals and targets, experimental activity, and data base sorting that allows for the creation of model variables used in the ANFIS modelling process These include training, validation, and testing using productivity assessment criteria such as Root mean square error (RMSE), mean square error (MSE), and coefficient-of-determination (R^2^)^37,60^.

### Evaluation of model performance

The effectiveness of the generated model was evaluated in order to confirm that it has a shown capacity for forecasting the target variables with a respectable level of accuracy. Based on relevant research, a number of performance requirements are derived from statistical measurements like R-values and less important function characteristics like MAE and RMSE using the equations in Eqs. (11) and (12).

11 $$RMSE = \sqrt {\frac{{\sum\limits_{i = 1}^{n} {\left( {E_{i} - M_{i} } \right)^{2} } }}{n}}$$

12 $$MAE = \frac{1}{n}\sum\limits_{i = 1}^{n} {\left| {E_{i} - M_{i} } \right|}$$

where n represents the number of data points in the study, $E_{i}$ is the actual or experimental results while $M_{i}$ is the model estimated values^61^.

## Results discussion and analysis

### Test materials characterization

Tables 1, 2 demonstrate the physical and chemical characteristics of the cement and admixtures (sisal fiber and aluminum scrap) used in this experimental study. The derived physicochemical characteristics indicate that AW possesses SiO_2_ (54.79%), Al_2_O_3_ (15.89%) and Fe_2_O_3_ (0.26%) producing a sum of 70.94% which revealed decent pozzolanic characteristics in agreement with ASTM C618, 98 requirements^24,52^. The abundance of CaO, Al_2_O_3_ and Fe_2_O_3_ at 11.3%, 20.6% and 6.405% respectively in the Portland limestone cement (PLC) component makes sure that the cement is fully hydrated, which enhances the concrete's mechanical strength behavior. The hydration compounds that are created when and aluminates silicon oxide from the admixture react with calcium to produce hard material over time. The results on the physicochemical behavior of test sisal fiber indicates aspect ratio, tensile strength, diameter and density of 250, 352Mpa, 1.1 mm and 1.6 g/cm^3^ respectively^62^. The chemical composition of the fiber showed 70% cellulose, 11% hemi-cellulose, 8% Lignin and 9% Pectin. The results of these tests on the compositions of admixtures are consistent with those of Bharath and Srikanth^20^.

**Table 1:** Physicochemical properties of cement and aluminum waste (source ^24^:).

Elemental oxide	AW (%)	PLC (%)
CaO	18.02	11.3
MgO	0.51	0.093
Fe_2_O_3_	0.27	6.405
Na_2_O	0.38	2.1
Al_2_O_3_	15.86	20.6
SiO2	54.78	52.4
ZnO	0.77	Trace
MnO	0.58	Trace
LOI	6.2	3.9
SO_4_	Nil	Trace
CdO	Trace	Trace
TiO_2_	Trace	0.52
CUO	Trace	Trace
K_2_O	Nil	2.6
Specific gravity	3.39	3.12
pH	8.2	8.8
Moisture content	0.28	2.4

**Table 2:** Physicochemical properties of sisal fiber (source ^20^:).

Properties	Results
Tensile strength (Mpa)	352
Elongation (%)	2.75
Diameter (mm)	1.1
Density (g/cm^3^)	1.6
Young’s modulus (Gpa)	18
Moisture content (%)	6.55
Aspect ratio	250
Specific gravity	0.73
Water absorption (%)	43.58

Chemical properties	% Present
Cellulose	70
Hemi-cellulose	11
Lignin	8
Ash	Nil
Pectin	9
Wax	2

**Figure 3:**
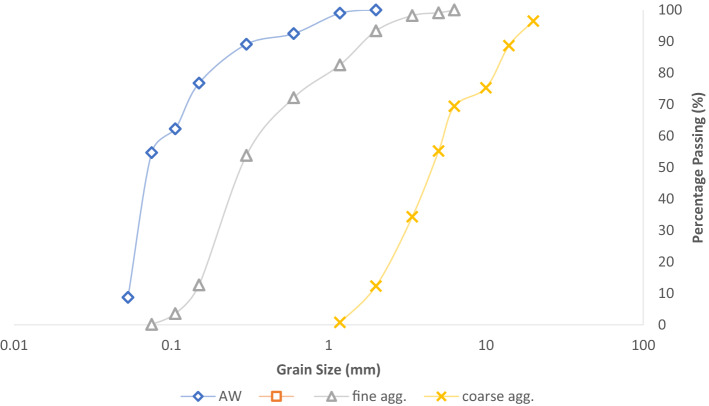
AW, fine and coarse aggregates sieve analysis.

A semi-log plot that displays the gradation and distribution of the aggregate components employed in the experimental inquiry is created from the sieve analysis of the test materials. From the data provided in Fig. 3, it can be seen that for the coarse and fine aggregate, correspondingly, 75.3–12.3% and 93.4–0.13% pass through sieve sizes of 10-2 mm and 2 mm-75 m. In the case of aluminum waste (AW), 100–54.68% of the material passes through filter sizes of 2 mm–75 µm. In addition, Table 3 presents the coefficients of gradation computation, which were derived using Eqs. (13) and (14). The findings achieved meet the BS 882 standards for increased concrete durability performance^63,64^.

13 $${\text{C}}_{{\text{u}}} = \frac{{{\text{D}}60{ }}}{{{\text{D}}10{ }}}$$

14 $${\text{C}}_{{\text{c}}} = \frac{{{\text{D}}30^{2} { }}}{{{\text{D}}60 \times {\text{D}}10{ }}}$$

**Table 3:** Gradation coefficients.

Test materials	D_10_	D_30_	D_60_	C_u_	C_c_
AW	0.054	0.065	0.105	1.94	0.75
Fine Agg	0.14	0.22	0.5	3.57	0.70
Coarse Agg	1.85	3	5.1	2.76	0.954

### Rheological properties results

#### Slump test results

**Figure 4:**
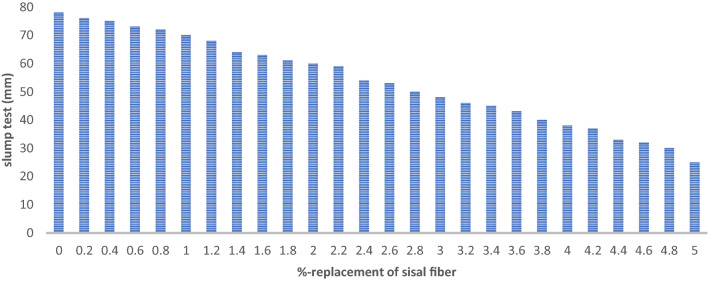
Slump test results.

Workability test result (Slump) which assesses the workability property of the concrete matrix indicates that value of the slump test reduces with increase in the AW content in the concrete mixture by requiring more water in order to make the mixture more workable in consonance with BS EN 12,350–2 specifications^65^. The high requirement of water is as a result of the presence of silica and increased surface area for the ash sample. This is because of silica-lime reaction require more water in addition to the water presented for the cement hydration reaction process. The obtained result is presented in Fig. 4 for the concrete samples replaced by 0–50% of sisal fiber and aluminum waste respectively^66^.

#### Setting time results

**Figure 5:**
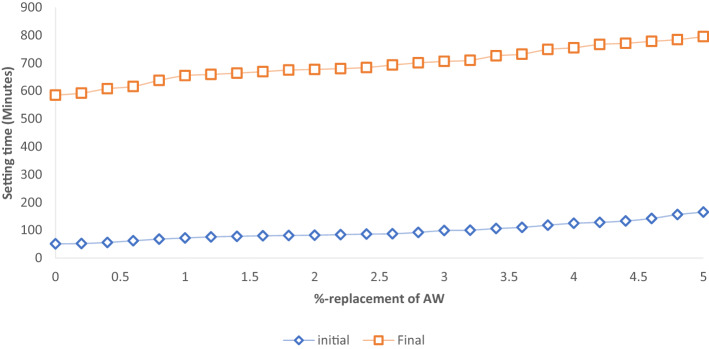
Setting time versus %-replacement of AW.

From the obtained results, it was perceived that the incorporation of AW effected the increase of the initial setting time from 51 to 165 min, while that of final setting time also rose from 585 to 795 min from 0–50% respectively. This is due to the fact that the inclusion of AW decreases the cement paste's surface area, which slows the hydration process and lengthens setting time. The low rate of heat generation results from the delayed hydration. This is crucial for mass concrete production since it calls for less hydration heat. The graph of cement/AW paste setting time versus %-replacement is presented in Fig. 5. As a consequence, the cement-AW paste's initial and ultimate setting times are longer as the AW concentration increases^67^.

### Mechanical strength response

The laboratory tests carried out to derive the mechanical behaviour of the blended concrete mixes were done on the hardened samples after 28 days hydration period. The compressive and flexural strength responses were calculated to determine the mixture combinations with maximum and minimum strength characteristics^68^.

#### Flexural strength

**Figure 6:**
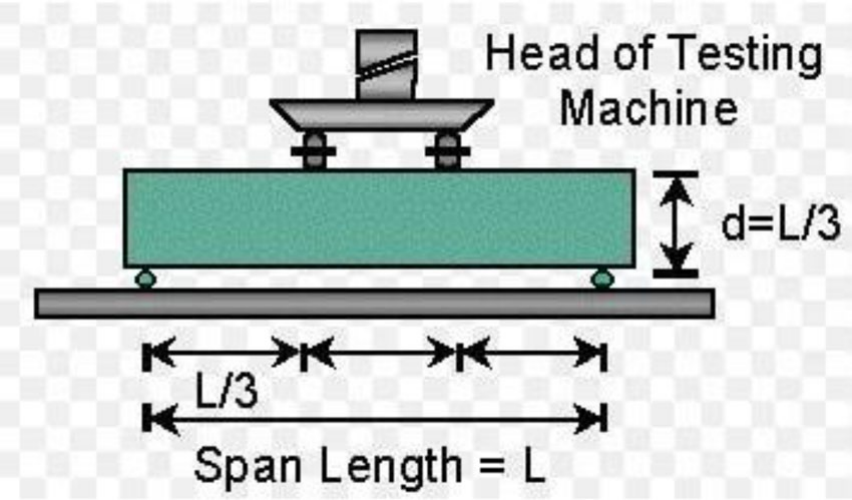
Loading configuration: four-point load flexural test (ASTM C78).

**Figure 7:**
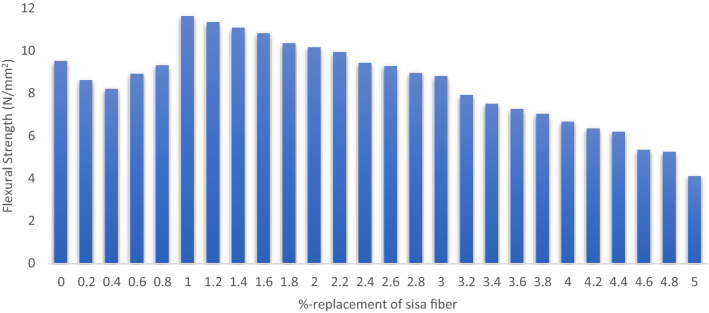
Flexural strength response.

The experimental methodology for the flexural strength tests is four point loading configuration flexural test according to ASTM C1161-02c(2008)e1 specification as shown in Fig. 6^57^. the cured blended green concrete beams of 100 mm × 100 mm × 400 mm with varying combinations of AW-sisal fiber mixtures were tested and the test results is presented in graphical chart in Fig. 7. Laboratory results indicated maximum flexural strength of 11.6 N/mm^2^ for 10% sisal fiber replacement while the minimum flexural strength of 4.11 N/mm^2^ was derived for 50% replacement. The calculated results showed that the addition of sisal fiber is effective at 0–10% replacement, however, from 20–50% replacement the strength properties tend to decrease linearly. This strength increment is due to the bonding and fiber integration in the concrete matrix which is also consistent with the findings of Alaneme and Mbadike^10^.

#### Test admixtures and compressive strength response interaction

**Figure 8:**
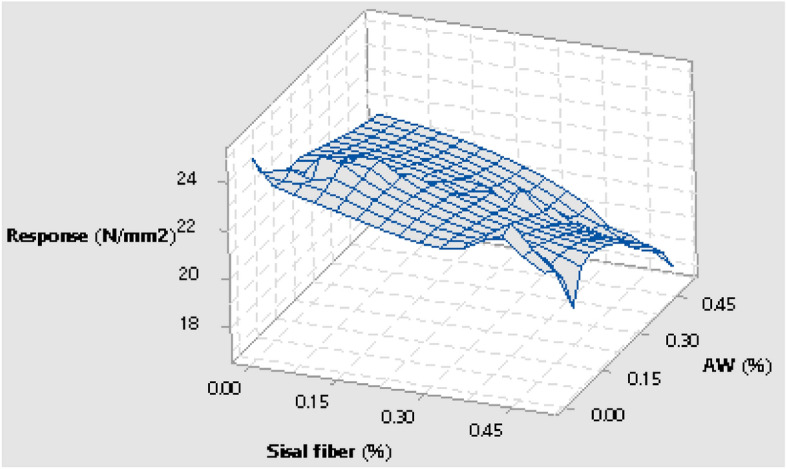
Concrete admixtures versus response.

The graphical interaction of the test admixture of sisal fiber and aluminium waste in respect to the concrete’s compressive strength response computed using Eq. (1) were presented in Fig. 8. The graph that is shown demonstrates how the optimal mechanical strength output was affected by the different AW and fiber quantities in the concrete mixture. The graphic 3-D surface plot shows that the highest compressive strength response was 24.97 N/mm2 when sisal fiber and AW were replaced at 0.08% and 0.1%, respectively. The lowest response was 17.02 N/mm2 when sisal fiber and AW were replaced at 0.5% and 0.45%, respectively. The experimental results obtained and the generated admixture ingredients’ fractions were taken to develop smart intelligent model using hybrid neuro-fuzzy inference system to evaluate the effects of these admixtures on the concretes strength performance. The computed experimental findings showed that the prepared green concrete performed more mechanically and was also within NCP 1^69^ and BS 8110: Part 1^70^ standards.

#### Experimental responses and system database for the ANFIS model evaluation

**Figure 9:**
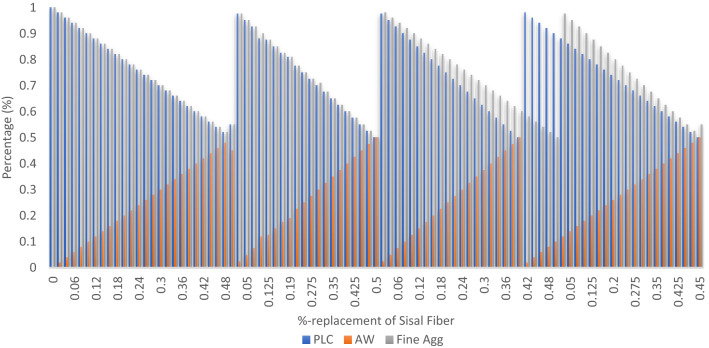
Input data.

**Figure 10:**
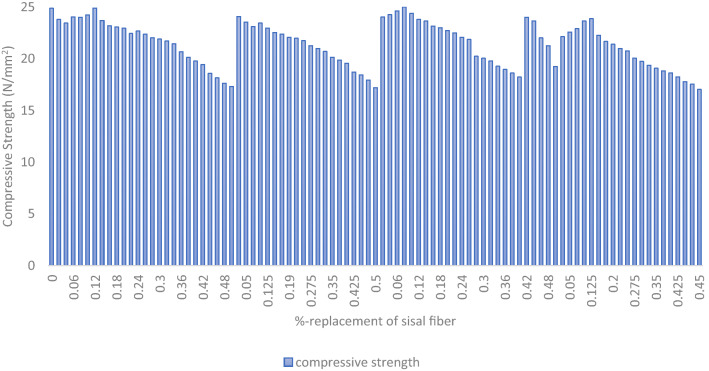
Output data.

Information derived from experimental findings, professional judgment, and pertinent literature that evaluates the mechanical-strength characteristics of created green fiber reinforced concrete. The model's input variables are blended mixtures of cement-AW and fine aggregates-sisal fiber combinations ranging from 0 to 50%, and its output variables are the concrete's mechanical strength qualities after curing for 28 days as seen in Figs. 9 and 10^71^. Table 4 displays the statistical findings of the output-input variables' correlation analysis, which highlights the linear interdependence between the parameters. The results indicates positive correlation coefficient for the PLC and fine aggregates fractions at 0.906 and 0.846 respectively with respect to the response variable. However, negative correlation coefficient score was calculated for the AW and sisal fiber fraction at − 0.906 and − 0.846 respectively. More so, Table 5 describes the data sets’ statistical characteristics and features in terms of its distribution, central tendency and variability^72^.

**Table 4:** Correlation statistical results of ANFIS model variables.

Variables	PLC	AW	Fine Agg	Sisal fiber	Targets
PLC	1				
AW	− 1	1			
Fine Agg	0.754115	− 0.75412	1		
Sisal fiber	− 0.75412	0.754115	− 1	1	
Compressive Str	0.905739	− 0.90574	0.845769	− 0.84577	1

**Table 5:** Statistical parameters of system datasets for the model development.

Variables	Mean	SE	SD	SV	Kurtosis	Skewness	Min	Max	Count
PLC	0.7421	0.0153	0.1458	0.0212	− 1.2068	0.0164	0.5	1	91
AW	0.2578	0.0153	0.1458	0.0212	− 1.2068	− 0.0164	0	0.5	91
Fine Agg	0.743	0.0152	0.1451	0.0211	− 1.2231	0.0295	0	0.5	91
Sisal Fiber	0.2569	0.0152	0.1451	0.0211	− 1.2231	− 0.0295	0	0.5	91
Compressive Str	21.4155	0.2288	2.1827	4.7640	− 1.0550	− 0.3200	17.02	24.97	91

*SE* standard error; *SD* standard deviation; *SV* sample variance.

### Smart intelligent model formulation

**Figure 11:**
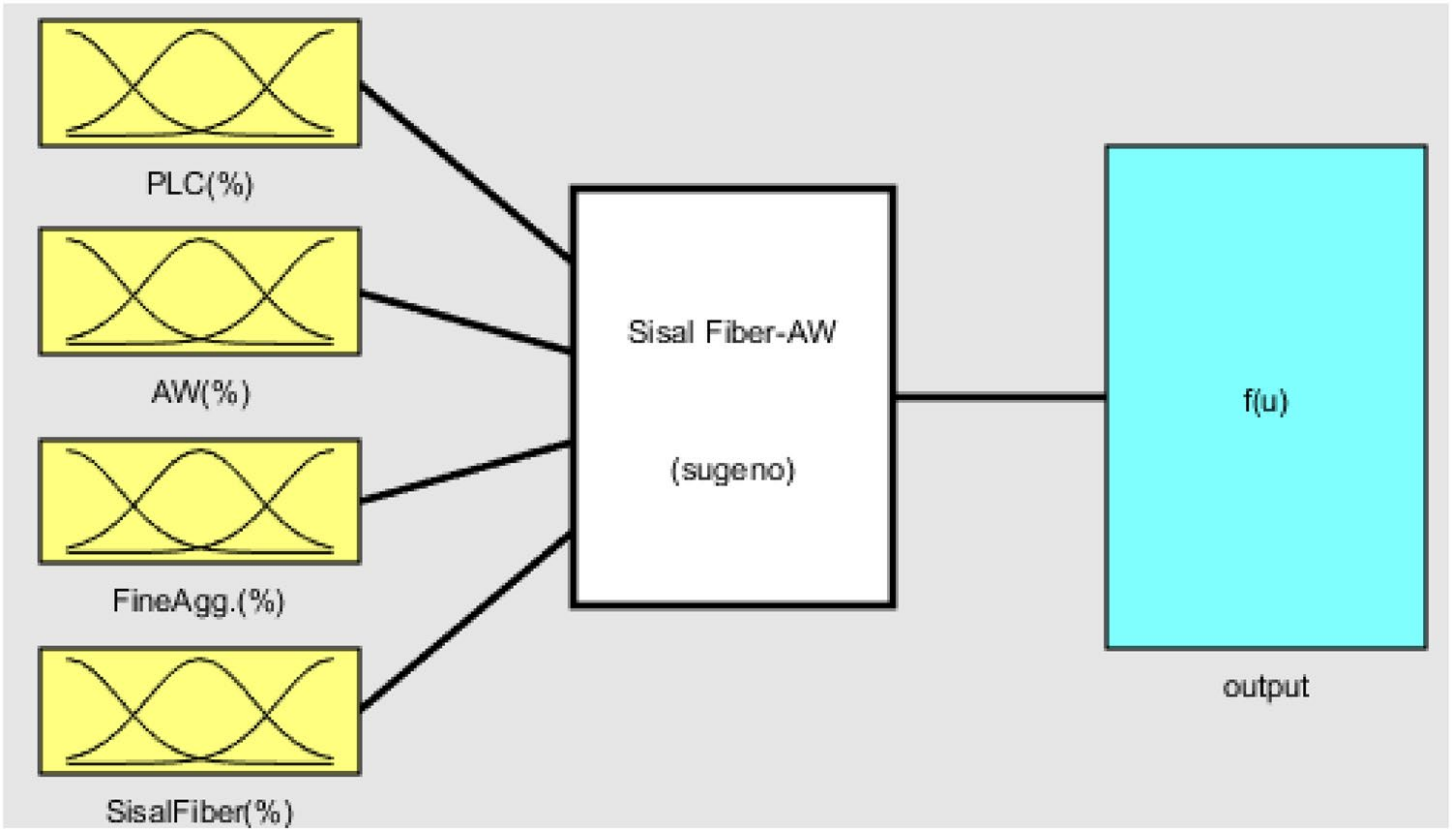
Connectivity of the ANFIS model parameters.

To properly develop the ANFIS model output-input variables, data from related published articles, experimental findings, and expert judgment were employed. Figure 11 displays the model variables' interaction demonstrating the input–output link. The mechanical behavior of green concrete generated with various ratios of AW and sisal fiber as admixtures was model-simulated, tested, trained, and validated using the ANFIS computation toolkit in MATLAB software. The system datasets were imported from the workspace and generated utilizing the Grid Partition Method of the Fuzzy Inference System (FIS), which would improve the accuracy of the data generalization^73^. Furthermore, the learning algorithm used uses a hybrid optimization technique that was put to use during the 100-epoch training of the fuzzy inference system (FIS). The processing settings are shown in Table 6, which includes the learning and membership function parameters for the data processing with zero error margin, 0.5 for range of influence, squash factor, reject, and accept ratios of 1.25, 0.15, and 0.5, respectively. This clever intelligent model building, as shown in Eq. (15), used the Gaussian membership function (gaussmf)^29^.

15 $$f\left( {x;\sigma ,c} \right) = e^{{\frac{{ - \left( {x - c} \right)^{2} }}{{2\sigma^{2} }}}}$$

where $\sigma$ and $c$ represents is the standard deviation and mean for the Gaussian function.

**Table 6:** ANFIS network processing parameter.

ANFIS network parameter	Settings
Fuzzy inference system type	Grid partition
Number of membership functions	3
Fuzzy rules numbers	81
Types of membership functions	gausianmf
Or method	Probor
Error margin	0
And method	Prod
Implication method	Minimum
Epochs	100
Aggregation	Maximum
Method of optimization	Hybrid
Defuzzification	Wtaver

#### Testing and training ANFIS

**Figure 12:**
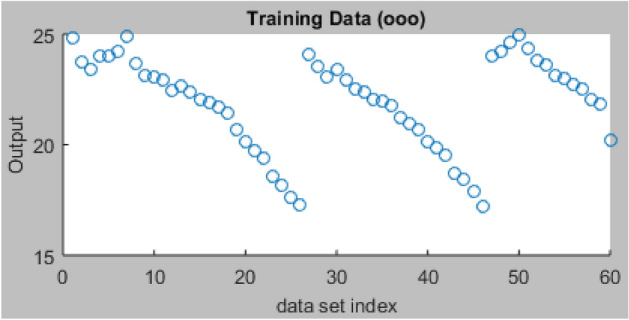
ANFIS model training graphical plot.

**Figure 13:**
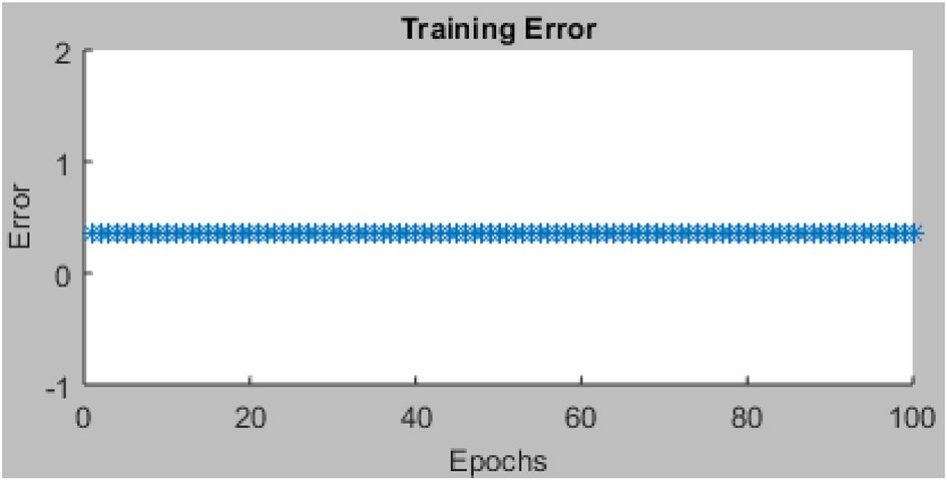
ANFIS training error plot.

**Figure 14:**
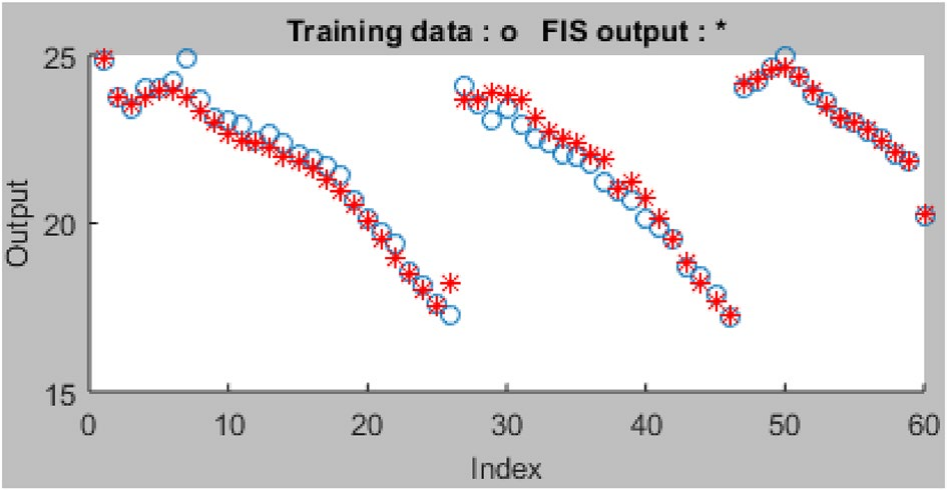
Training data plots.

Using the recommended hybrid optimization training techniques and FIS variables, ninety-one (91) datasets that are employed for the modelling process are split and sorted into 2 parts to accomplish the network's training (60) and testing (31), respectively. Figure 12 displays the datasets imported from the workspace for the ANFIS network training, which contain one output and four input variables. It also displays the graphic plot of the 60 index for the network training. After 28 days of curing, the ANFIS network was trained using the loaded datasets to accurately generalize the data and assess the compressive strength properties of the AW-sisal fiber concrete. For this operation, the hybrid training approach was used with settings for 100 Epochs, 0% tolerance, and grid partitions. After the training procedure, a training error result of 0.365 was produced, indicating improved performance as shown in Fig. 13. As shown in Fig. 14, after the ANFIS network training using the sorted experimental data is complete, the 60 data points that were previously empty and blue are highlighted with red asterisks to show accomplishment of the training stage. The average testing error was 0.3648^74^.

**Figure 15:**
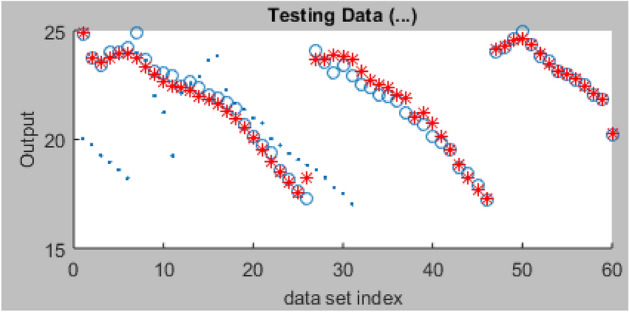
Plot of ANFIS training and testing data sets.

Following the completion of training, the 31 datasets reserved for testing the trained model are further imported from the MATLAB program workspace to guarantee improved predictive accuracy. As illustrated in Fig. 15, the loaded testing datasets and the trained ANFIS network datasets were displayed together. The loaded testing datasets were colored blue with 31 index points.

**Figure 16:**
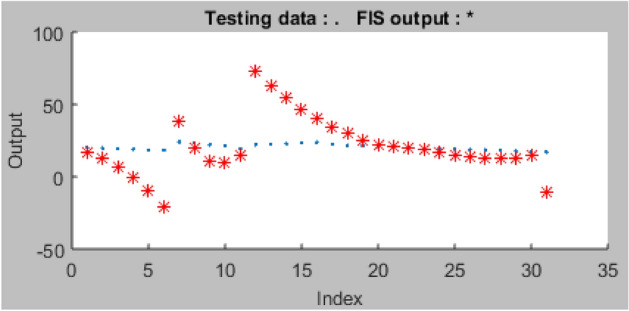
Plot of ANFIS testing datasets.

In order to assure a better outcome in terms of predictive performance, the network testing was conducted using the processing parameters originally set out for the ANFIS model training. Using the set-out datasets, a testing error of 2.663 was computed after the testing, as shown in Fig. 16.

#### Constructed network architecture of ANFIS

**Figure 17:**
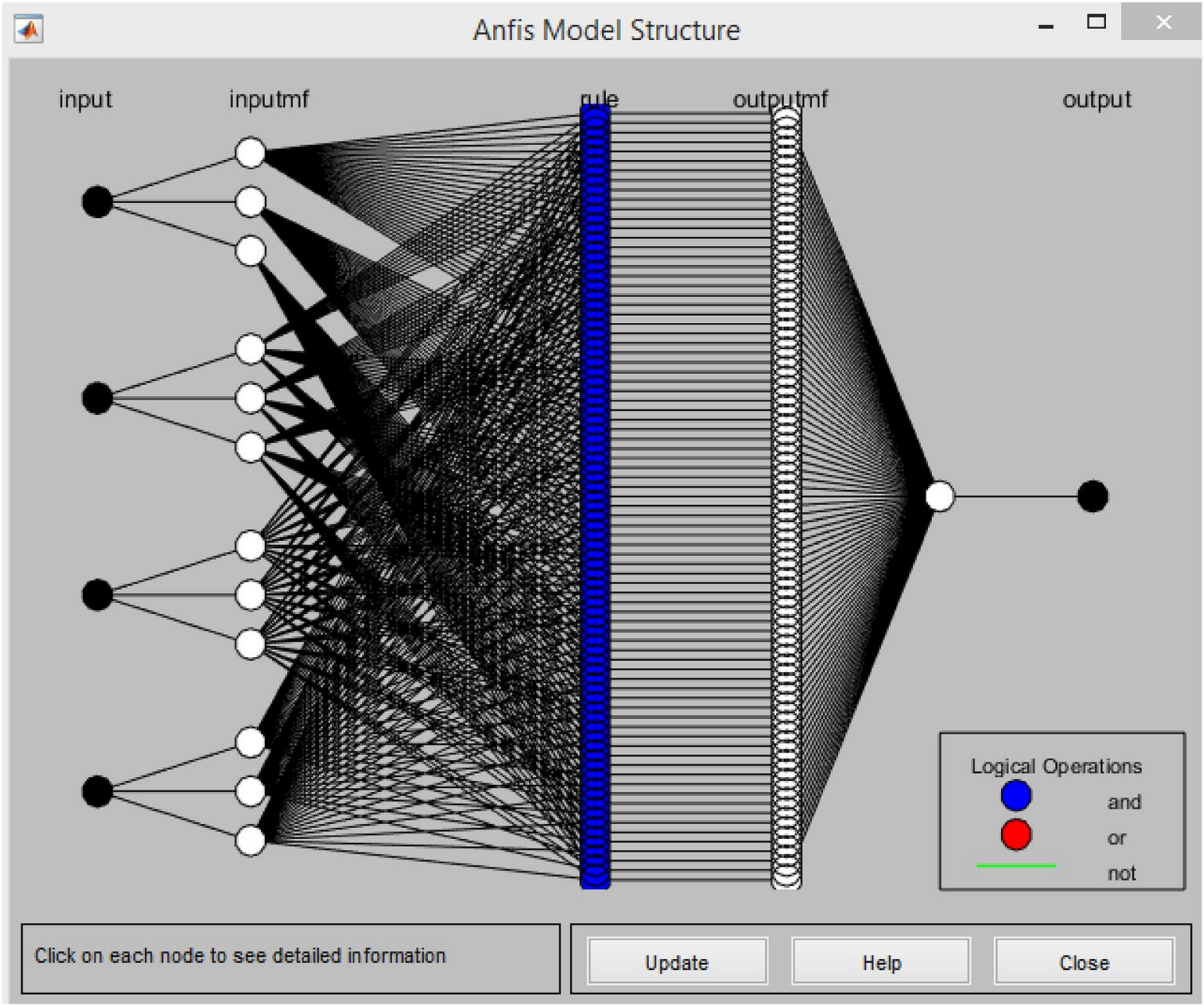
ANFIS model structure generated from MATLAB R2020a.

Figure 17 as generated from the MATLAB R2020a software depicts the ANFIS architecture produced after training and testing using the datasets provided to the network. The intricate interaction between the input variables, the fuzzification node, the input signal weight aggregation, normalization of the network's firing intensity, fuzzy if–then rule creation, and total cumulative function for the output nodes are shown. The design combines four input parameters, PLC (%), AW (%), fine aggregates (%), and sisal fiber (%), with one output for the compressive strength response of the concrete^75^.

#### ANFIS network membership function plots

**Figure 18:**
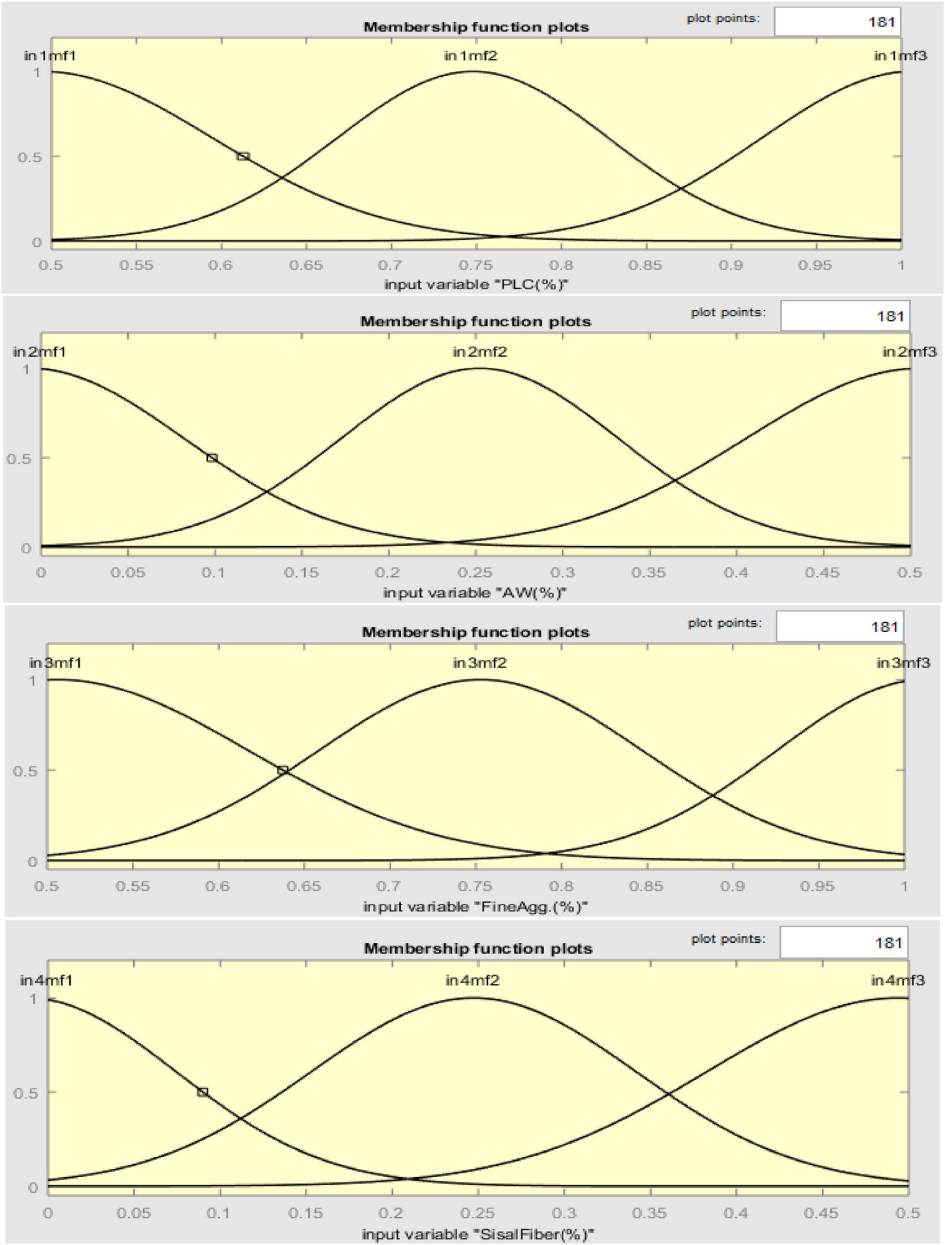
Membership function plots of model variables.

The membership functions (MF) plots for the model input variables for the developed ANFIS network is generated automatically after the training and testing processes as shown in Fig. 18. The plot indicates data range for input parameters on the x-axis against the universe of discourse which possess values ranging from 0 to 1 on the y-axis^76^.

#### ANFIS-model variables graphical expression

**Figure 19:**
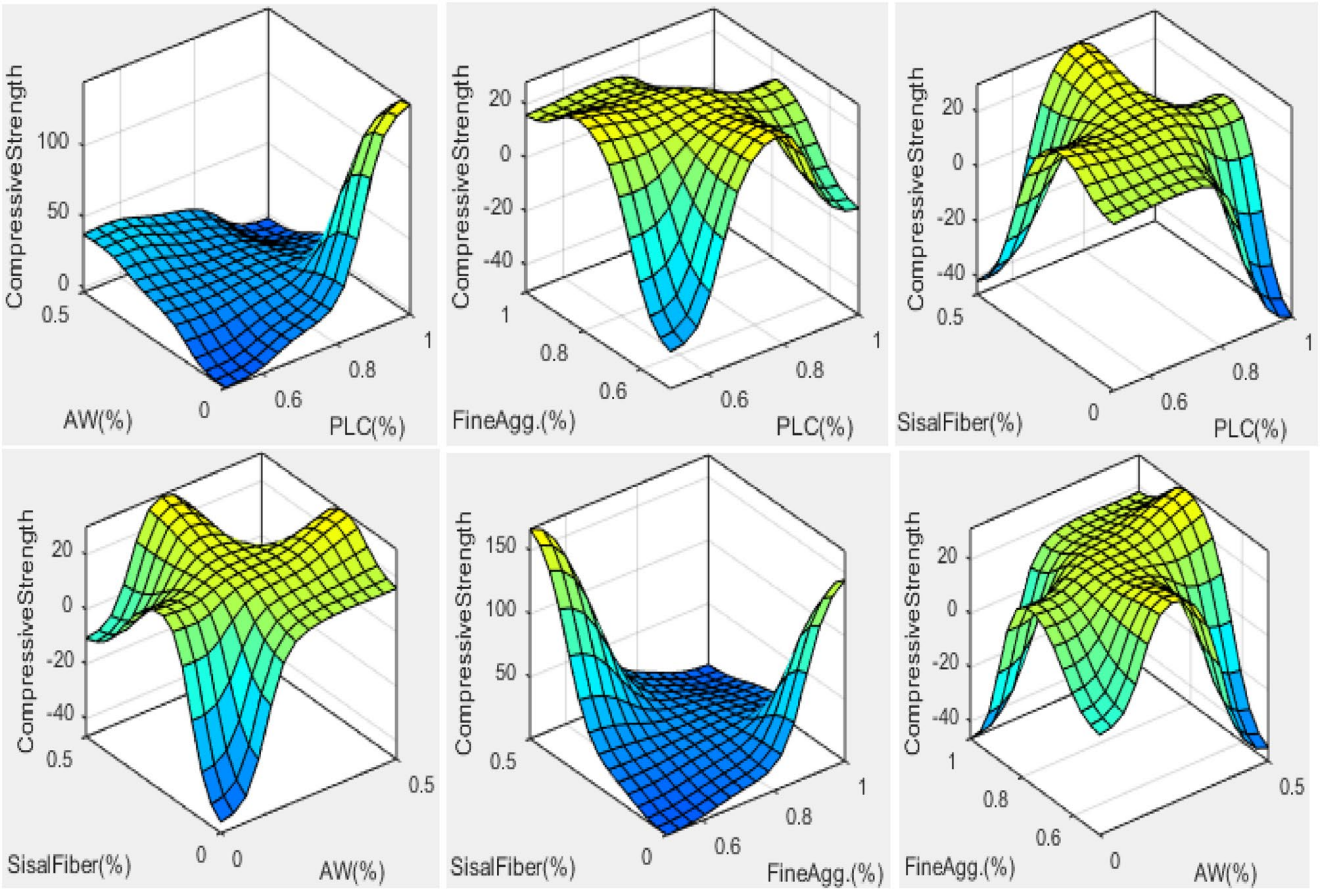
Model variables’ 3D-surface plots.

**Figure 20:**
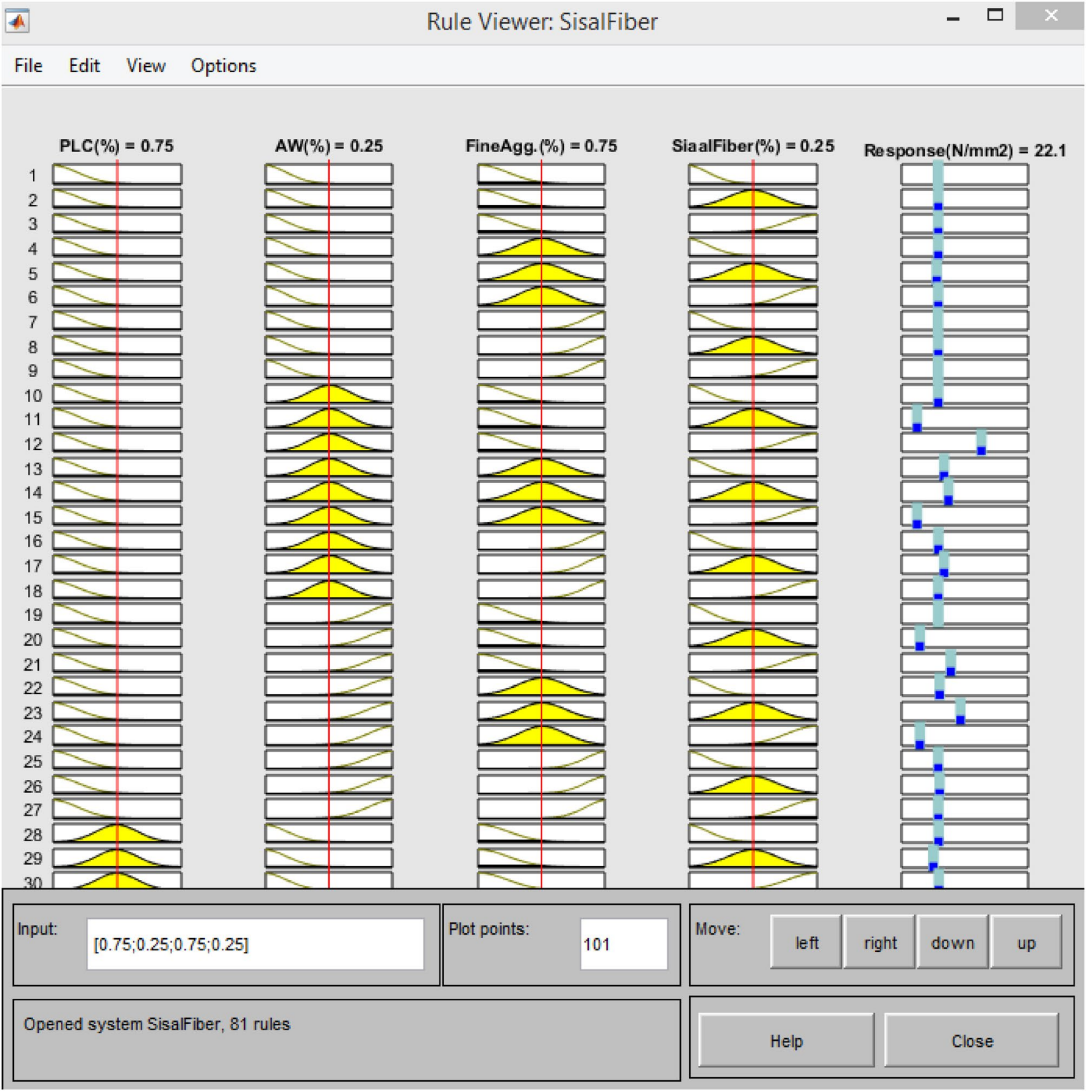
Rule viewer generated from MATLAB R2020a.

The ANFIS model, which was used to evaluate the compressive strength qualities of concrete made with AW-sisal fiber, uses a hybrid learning strategy to learn how to generalize the data sets that are provided to it. A given input space may be correctly mapped by the created smart model to the appropriate output response. The connections between the model variables are assessed to determine their individual significant relevance or impacts, as illustrated in Figs. 19, 20, using a three-dimensional surface plot and simulation rule viewer. The plots show that addition of sisal fiber and aluminum waste in the matrix to substitute fine aggregates and cement from 0–45% and 0–25% respectively resulted to enhancement in the compressive properties of the green concrete produced. After data sets have been made more generic by training and testing, the rule viewer will make it possible to derive simulated ANFIS model outputs. To determine the ideal ratios of the concrete mixture to accomplish sustainable building, the impacts of sisal fiber and aluminum waste integration in concrete were evaluated^77^.

#### Model validation

**Figure 21:**
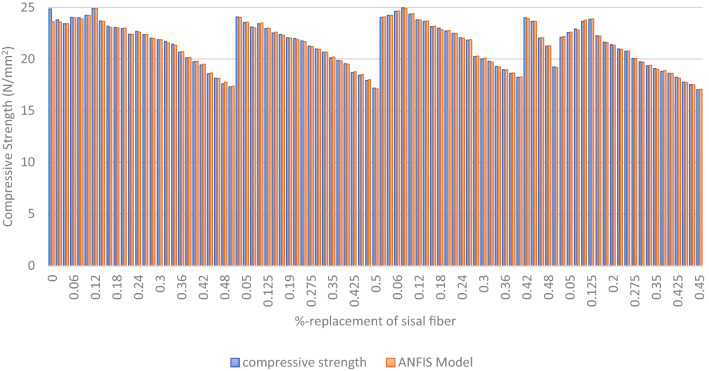
Experimental and ANFIS model results.

**Figure 22:**
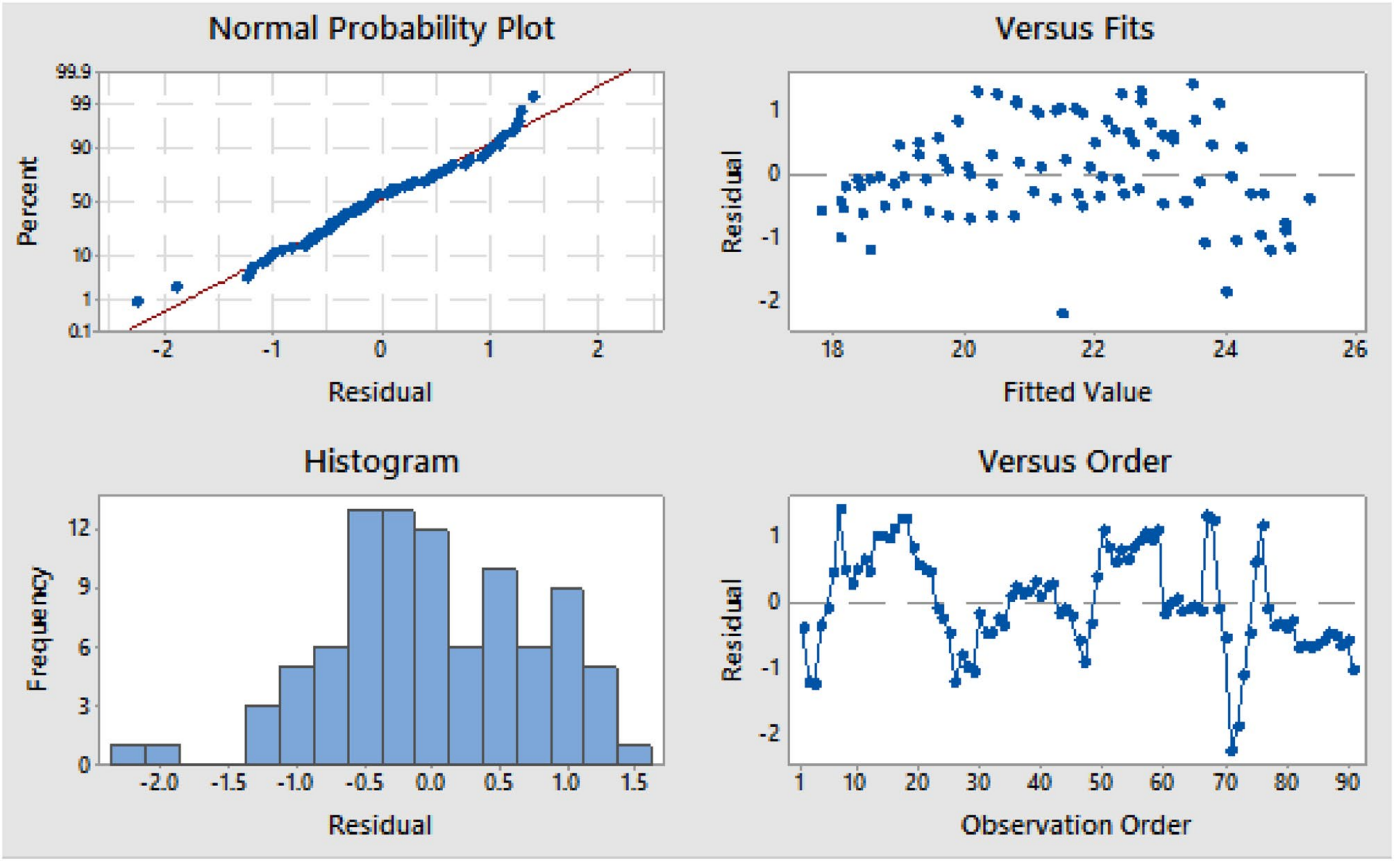
Residual plots for the target response using MLR.

The ANFIS approach was used in the modeling process to optimize the experimental findings acquired to assess the compressive strength characteristics of AW-sisal fiber concrete, and the model result is shown in a graphical chart shown in Fig. 21^78^. Additionally, using Minitab 18 statistical software, the generated ANFIS model's performance in terms of prediction accuracy was evaluated by statistically comparing the real or experimental findings with those of the ANFIS model. MLR statistics, RMSE, MAE, and coefficient of determination were used in this comparison. In Table 7 and Fig. 22, the MLR findings and residual graphs are shown. The R-sq. (adj.) and R-sq. values of 88.16% and 88.69%, respectively, indicated that the compared datasets had an average degree of correlation based on the results of the MLR calculation^61,79^.

**Table 7:** Multiple linear regression statistical results.

Model summary			Regression coefficients				
S	R-sq. (adj.)	R-sq	Constant	PLC	AW	Fine Agg	Sisal fiber
0.751	88.16%	88.69%	59.0	− 10,121	− 10,131	10,088	10,082

According to the derived statistical findings, which are represented in Table 8 by MAE, RMSE, and coefficient of determination values of 0.1318, 0.412, and 99.57%, correspondingly, there is no appreciable discrepancy between the experimental or real data and the results of the ANFIS model. The result suggests that the created ANFIS model performed better than the linear regression model as well. The model's quantitative assessment results concurred with the conclusions of Alaneme et al*.*^34^ and Wang et al*.*^80^.

**Table 8:** Performance evaluation of ANFIS model.

Target output	Statistical parameter	Requirements	Calculated results	Remarks
Compressive strength	MAE	Close to 0	0.1318	Excellent
	RMSE	Close to 0	0.412	Very good
	R^2^	Greater than 0.8	0.9957	Very good

**Figure 23:**
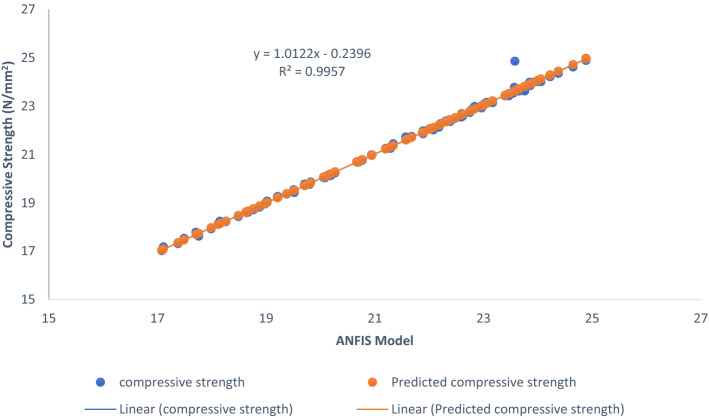
ANFIS model line of fitness plot.

Figure 23 displays the slope of the regression line comparing laboratory or real findings to the outcomes of the ANFIS model. The line of best fit, which is the straight line that accurately predicts the given sets of data, is shown in the figure. In Eqns. 16 is given the line-of-fit equation for the compressive strength response of the AW-sisal fiber concrete.

16 $$y = 1.0122x + 0.2396$$

## Conclusions

For the modeling of the mechanical strength properties of concrete with fine particles and cementitious fraction partially replaced by aluminum waste and sisal fiber, correspondingly, from 0 to 50%, adaptive neuro-fuzzy inference system (ANFIS) was used. The research study's findings allow for the following deductions:

- The concrete ingredients passed testing for the essential chemical and physical characteristics for excellent pozzolanic performance. The findings of the particle size distribution highlighted the necessity for strong gradation features to create concrete with enhanced durability properties. Following a 28-day hydration interval, concrete's compressive strength findings were also obtained. The computed experimental findings show that sisal fiber and AW were replaced to a maximum compressive value of 24.97 N/mm^2^ at 0.08% and 0.1%, respectively, and to a minimum compressive value of 17.02 N/mm^2^ at 0.5% and 0.45%, respectively.
- The flexural strength test response of the fiber concrete indicated maximum strength of 11.6 N/mm^2^ for 10% sisal fiber replacement while the strength reduces with increase content of sisal fiber in the concrete matrix with minimum flexural strength of 4.11 N/mm^2^ derived for 50% replacement.
- The slump results showed that value of the slump test reduces with increase in the AW content in the concrete mixture by requiring more water in order to make the mixture more workable. The setting time test results on the cement-AW blend showed that the addition of AW effected the increase of the initial setting time from 51 to 165 min, while that of final setting time also rose from 585 to 795 min from 0–50% respectively.
- For the creation of the model, the system database was comprised of the sorted information obtained through the experimental approach. The 91 datasets in total were separated into two groups, with the latter group receiving 60 datasets and the former group receiving 31 datasets, respectively, in order to test and train the adaptive network.
- Additionally, MLR, MAE, RMSE, and r^2^ were used to evaluate the generated ANFIS model's ability to predict outcomes. The findings of the statistical analysis show an MAE of 0.1318, RMSE of 0.412, and R2 of 99.57%, while the MLR model shows a coefficient-of-determination of 88.69%. Incorporating the industrial waste residues under investigation in this study offers a viable alternative to the traditional concrete ingredients for the production of green concrete that reduces the amount of carbon dioxide released during the manufacture and use of cement, the cost of infrastructural construction materials, the disposal of industrial waste without regard for its intended use, and other factors that contribute to sustainability and the environment.
- Furthermore, smart-intelligent model development was used to assess the created mechanical strength results of concrete reinforced with AW-sisal fiber. This was accomplished via the use of ANFIS soft computing. The findings of this research demonstrate the versatility of the ANFIS approach with relation to its use in modeling the engineering behavior of cement-additive concrete blends for sustainability.

## Data Availability

All data generated or analyzed during this study are included in this published article.

## Abbreviations

ANFIS: Adaptive neuro-fuzzy inference system
AW: Aluminum waste
AI: Artificial intelligence
ANN: Artificial neural networks
r^2^: Coefficient of determination
FIS: Fuzzy inference system
guassmf: Gaussian membership function
MF: Membership function
MLR: Multiple linear regression
probor: Probabilistic OR
RMSE: Root mean squared error
MAE: Mean absolute error
MSE: Mean squared error
SCM: Supplementary cementitious materials
SF: Sisal fiber
